# Perioperative Urinary Catheter Use and Association to (Gram-Negative) Surgical Site Infection after Spine Surgery

**DOI:** 10.3390/idr15060064

**Published:** 2023-11-10

**Authors:** Alexandre Ansorge, Michael Betz, Oliver Wetzel, Marco Dimitri Burkhard, Igor Dichovski, Mazda Farshad, Ilker Uçkay

**Affiliations:** 1 University Spine Centre Zürich, Balgrist University Hospital, University of Zurich, 8008 Zurich, Switzerland; 2 Unit for Clinical and Applied Research, Balgrist University Hospital, University of Zurich, 8008 Zurich, Switzerland; 3 Infectiology and Infection Control, Balgrist University Hospital, University of Zurich, 8008 Zurich, Switzerland

**Keywords:** spine, surgical site infection, urinary tract infection, urinary catheters

## Abstract

This study evaluates potential associations between the perioperative urinary catheter (UC) carriage and (Gram-negative) surgical site infections (SSIs) after spine surgery. It is a retrospective, single-center, case-control study stratifying group comparisons, case-mix adjustments using multivariate logistic regression analyses. Around half of the patients (2734/5485 surgeries) carried a UC for 1 day (median duration) (interquartile range, 1–1 days). Patients with perioperative UC carriage were compared to those without regarding SSI, in general, and Gram-negative, exclusively. The SSI rate was 1.2% (67/5485), yielding 67 revision surgeries. Gram-negative pathogens caused 16 SSIs. Seven Gram-negative episodes revealed the same pathogen concomitantly in the urine and the spine. In the multivariate analysis, the UC carriage duration was associated with SSI (OR 1.1, 95% confidence interval 1.1–1.1), albeit less than classical risk factors like diabetes (OR 2.2, 95%CI 1.1–4.2), smoking (OR 2.4, 95%CI 1.4–4.3), or higher ASA-Scores (OR 2.3, 95%CI 1.4–3.6). In the second multivariate analysis targeting Gram-negative SSIs, the female sex (OR 3.8, 95%CI 1.4–10.6) and a UC carriage > 1 day (OR 5.5, 95%CI 1.5–20.3) were associated with Gram-negative SSIs. Gram-negative SSIs after spine surgery seem associated with perioperative UC carriage, especially in women. Other SSI risk factors are diabetes, smoking, and higher ASA scores.

## 1. Introduction

During or after spine surgery, anesthesiologists, surgeons, and nurses often turn to perioperative urinary catheters (UCs) for many reasons: postoperative urine retention, discomfort, immobility, incontinence, frailty, and danger of sacral decubitus [1,2,3]. However, UC use is a well-established risk for a symptomatic urinary tract infection (UTI). According to a nationwide Swiss prevalence study in 2004, as well as other prevalence studies from other European countries, UC use is responsible for at least 60% of all symptomatic UTI among hospitalized patients [4]. Furthermore, pilot studies and clinical experience suggest that perioperative UC use might not only be associated with UTI, but might also lead to more (Gram-negative) surgical site infections (SSI) of presumed urinary origin [5]. Interestingly, several author groups advocate the existence of a microbiological gradient with an increasing proportion of Gram-negative pathogens when descending from the cervical to the sacral spine [6,7]. If there is truly a link between UC use and SSIs, one could tailor specific interventions to prevent UTI and (Gram-negative) SSIs [8]. In this study, we investigate the association of UC and UTI with deep SSIs and especially with deep Gram-negative SSIs [9,10].

## 2. Materials and Methods

### 2.1. Setting and Perioperative Antibiotic Prophylaxis

The authors’ institution is a tertiary center for orthopedic surgery in Switzerland. Since 2014, we have invited all adult patients to sign a general consent allowing us to register healthcare data for retrospective studies (laboratory, demographics, therapy, and co-morbidities). The standard systemic perioperative antibiotic prophylaxis for spine surgery at the authors’ institution is 1.5 g cefuroxime administered intravenously, which is doubled to 3.0 g for patients ≥ 80 kg [11]. In case of cefuroxime intolerance, clindamycin (600 mg to 900 mg) or vancomycin is used. The duration of prophylaxis remained unchanged throughout the study period and consisted of three parenteral doses of the same agent (twice for vancomycin). Local prophylactic antibiotics are avoided, except for local vancomycin powder in revision and scoliosis surgery. The insertion of urinary UCs in the perioperative period is avoided, except in case of surgeries with planned duration of >2 h, postoperative urinary retention, perineal maceration leading to a sacral decubitus, urinary incontinency, severe postoperative pain, and patients’ immobility (according to the in-house checklist). The Laboratory of Microbiology (Institut für Medizinische Mikrobiologie, USZ, in Zurich) processed all microbiological cultures according to the usual Clinical and Laboratory Standards Institute (CLSI) criteria.

### 2.2. Study Criteria and Data Collection

All spine surgeries performed in adult patients at the authors’ institution between 1 January 2014 and 31 October 2020 were included. The database was closed on 7 December 2021. Hence, the minimal follow-up time after the last inclusion was thirteen months. Episodes with open fractures, other orthopedic surgeries, spine infections without prior index surgery, and pediatric cases were excluded. Deep SSIs were defined in line with necessity for unplanned surgical revision and the presence of the same bacteria in several intraoperative tissue specimens, including in pseudarthrosis-related occult infections [12,13]. For this study, superficial SSIs that were not revised in the operating theater were not included [9]. All SSI diagnoses were independently re-confirmed by an Infectious Diseases physician (IU) with long experience in orthopedic infection. A UTI was defined by the presence of ≥10^4^ bacterial colonies/mL in the urine together with compatible symptoms, for which the patient was treated with antibiotics. The congruence of the urinary pathogens with those of SSI was based on bacterial species and the corresponding antibiotic susceptibility patterns. A medical informatician (P.J.) composed the database from the hospital’s informatic patient information system. He selected important risk factors for SSI: age, weight, height, body mass index (BMI), American Society of Anesthesiologists (ASA)-Score, diabetes, date, type and localization of index surgery, duration of surgery, date and indication for revision surgery. This database was cleansed and merged with others from the Unit for Clinical and Applied Research. Regarding the literature search in German and English languages, two authors (A.A. and I.U.) screened for publications in PubMed and the internet using combinations of the MeSH terms “spine”, “surgery”, “infection”, “urine”, and “catheter”. They also searched for further articles through the retrieved papers. We excluded papers indicating UTIs without SSI.

### 2.3. Statistical Analyses

The primary outcome was the association of spine SSI with perioperative UC use during the index surgery. Secondary outcome parameters were the possible link between SSI and symptomatic UTI after the index surgery; risk factors for Gram-negative SSI specifically; variables associated with perioperative UC use; and the proportion of UTI pathogens resistant to the perioperative antibiotic prophylaxis during the index surgery. Of note, we assessed the role of UCs in Gram-negative SSI twice: the first time within our cohort of all spine surgeries, the second time comparing Gram-negative vs. Gram-positive SSIs among all internal and external SSIs. Hence, for the evaluation of risk factors for Gram-negative SSI, we increased the sample size by adding transferred patients with infections that had been operated on previously elsewhere.

Groups were compared with the Pearson-χ^2^ (categorical variables) or the Wilcoxon-ranksum-test (non-parametric, continuous variables). Multivariate logistic regression analyses with the outcome SSI were adjusted for the large case-mix. Independent variables with a *p*-value ≤ 0.05 in univariate results were stepwise introduced in the multivariate analysis, while we always fitted urinary variables into the multivariate model. We checked for collinearity and interaction and included, at minimum, 9–10 outcome events per predictor variable. The final regression model was composed of SSI, UC, UTI, diabetes, age, duration of surgery, ASA-Score, weight, and BMI. We renounced on a formal propensity score matching on UC use, because the indication for its use was too frequent and arbitrary. Instead, we controlled for UC use performing separate multivariate analyses and stratifications. We used STATA™ (15.0, College Station, TX, USA) and considered *p*-values ≤ 0.05 (two-tailed) as significant.

## 3. Results

### 3.1. General Results

We included 5485 of our own spine surgeries and added 97 external SSIs for the secondary outcome “Gram-negative SSI versus Gram-positive SSI”. Overall, 2782 episodes (51%) of 5485 were documented among females and 600 episodes (11%) among diabetic patients. The median age, BMI, and ASA-Score of the patients were 63 years, 26.7 kg/m^2^, and 2 points, respectively. The compliance with the institutional antibiotic prophylaxis was >95% [1]. The lumbar spine was most often involved in surgery (4382 episodes; 80%), followed by the cervical (11%) region, surgeries involving more than one spinal region (6%), and the thoracic region (3%). Overall, 47% were implant-related surgeries and 32% revision surgeries. The median operation time was 78 min (interquartile range, 62–122 min). The median blood loss per operation was 200 mL and the median length of hospital stay was 5 days. The surgery notes identified 11 different main surgeons (first operators) having performed the surgeries.

### 3.2. Use of Urinary Catheters and Associations with Surgical Site Infections

Perioperatively, patients in approximately half of the surgeries (2734 episodes among 5485 surgeries) received a UC for a median duration of 1 day (interquartile range, 1–1 d). In 328 episodes (328/5485; 6%), the clinicians sampled a microbiological urinary culture analysis (patients with or without UC). In the group comparison, patients with substantial co-morbidities, revision surgeries, implant surgery, diabetes, high ASA-Scores, long operations, women, aged, and adipose persons yielded significantly more frequent UC carriage than other patients (Table 1).

The total deep SSI risk was 1.2% (67 SSIs among 5485 surgeries). Each SSI episode was microbiologically confirmed by a median of five intraoperative tissue samples, and 28 of them were additionally confirmed by histology. Sixteen (16/67; 24%) SSI episodes were caused by Gram-negative bacteria, of which seven yielded the same pathogen concomitantly in the urine and the spine. The four most frequent causative pathogen (groups) of SSI were coagulase-negative *staphylococci* (39%), *Cutibacterium acnes* (27%), *Staphylococcus aureus* (21%), and *Escherichia coli* (9%). The three most frequent Gram-negative pathogens were *E. coli* (9%), *Klebsiella* spp. (3%), and *Proteus* spp. (3%). Overall, 10 SSIs (15%) were polymicrobial, including 42 different microbiological constellations (including skin commensals). Table 2 resumes the comparison between spine surgeries with SSI and without. In total, 174 episodes (174/5485; 3%) were accompanied by symptomatic UTI, of which 40% of the urinary pathogens were resistant to the prior prophylaxis (cefuroxime) during the index surgery. We witnessed no episodes of clinical pyelonephritis. The most frequent prophylaxis-resistant bacterial group in the urine were Gram-negative rods such as *Enterobacter* spp. or *Pseudomonas* spp., followed by enterococci. All symptomatic UTIs were treated by a targeted systemic antibiotic therapy (range of duration 3 to 10 days) and were ultimately classified as cured from UTI. No patient died of urosepsis or UTI.

### 3.3. Multivariate Adjustments

In the multivariate analysis for the outcome “overall SSI” (Table 3, left), the duration of UC carriage was significantly associated with SSI (odds ratio 1.1, 95% confidence interval 1.1–1.1), albeit less significantly than with other classical risk factors such as diabetes (OR 2.2, 95%CI 1.1–4.2), smoking (OR 2.4, 95%CI 1.4–4.3), the ASA-Score (OR 2.3, 95%CI 1.4–3.6), revision surgery, or long operation times. In contrast, the lumbar site (OR 0.6, 95%CI 0.3–1.2), age, or female sex were not associated with an overall higher SSI risk. Regarding the association of UCs specifically with Gram-negative SSIs, we performed a second analysis in a patient population of 134 SSI episodes (Table 3, right), among which 27 (20%) were due to Gram-negative pathogens. In the corresponding multivariate regression, women (OR 3.8, 95%CI 1.4–10.6) and a UC carriage beyond 1 day (OR 5.5, 95%CI 1.5–20.3) remained associated to Gram-negative SSIs. Our models showed an insignificant goodness-of-fit, and the Receiver-Operating-Curve (ROC) values were 0.90 and 0.79, respectively, highlighting a good accuracy of the final statistical models.

## 4. Discussion

In this single-center case-control study, UC after elective spine surgery was associated with both the occurrence of SSI and the stratum of Gram-negative SSI, in particular. Especially the duration of UC use beyond 1 day, and the female sex were statistically associated with Gram-negative SSI. The clinical relationship is plausible because traditionally women reveal a higher risk for symptomatic UTI [14]. However, we cannot assess the pathophysiological mechanisms with which potential urinary pathogens would move into the operated spine; there is no direct contact between the spine surgeons and the genital area of the patients during operation, and the risk of a hematogenous infection is rather low in the entire field of orthopedic surgery [15]. Therefore, we presume that contamination might occur via the hands of healthcare workers nursing the genital region, managing the UCs, and changing the dressings [7,14,16]. This contamination via the hands is considered as the most likely cause of a postsurgical acquisition of SSI [9,17,18]. According to other author groups, the association between UTI and SSI probably has a more complex, non-identified, and multifactorial explanation, as it cannot be inferred that UTI is a direct risk factor for SSI in all cases [5].

Regarding our other predictors of spinal SSIs, such as a high BMI, ASA-Score, or active smoking, all are entirely established in existing spine literature, and deny an important selection bias for our study population [8,19,20,21,22]. Our SSI risk of 1.2% is lower than most incidences reported and is slightly better than the 1.5% that we already published in 2018 [5,13,19,21,23,24]. For example, a recent meta-analysis including 22,475 spine patients computed a pooled SSI risk of 3.1% [23].

The literature is sparse when linking UC and symptomatic UTI to SSI after spine surgery (Table 4).

Even large reviews targeting SSI prevention do not even discuss or mention the potential danger stemming from prolonged UC use [8,20,22,24,27,28,29]. Only three studies exclusively report a possible link of postoperative UC carriage, or symptomatic UTI, with SSI. The first is a French multicenter observational prospective study published in 2012 [25]. It included 169 cases and showed a prolonged duration of UC carriage greater than five days to be associated with SSI on univariate analysis. No multivariate analysis was performed due to underpowering. The second paper stems from the same research group [13]. It included 518 cases and found the presence of a UC again associated to SSI on univariate analysis, but failed to demonstrate that UC could be an independent risk in the multivariate analysis. The third stems from a Spanish group and was published in 2014 [5]. It confirms our findings. The authors retrospectively analyzed 466 patients after instrumented spine surgery [5]. Eighty-nine patients yielded a UTI (89/466; 19%), 54 reported an SSI (54/466; 12%), and 22 had both infections (22/466; 5%), of which nine (9/22; 41%) were caused by the same microorganism. In their study, the urinary tract was the probable source of SSI by Gram-negative bacteria in 38% (8/21) of cases. Their SSI risk was 24.7% (22/89) in patients with confirmed UTI and 8.5% in those without UTI (32/377), which is far beyond the habitual incidence in the literature [23]. On their multivariate logistic regression analysis, UTI was significantly associated with SSI (OR 3.1, 95% CI 1.6–6.1). Moreover, patients receiving ciprofloxacin for UTI had higher microbial resistance risk to quinolones at SSIs (46%) than those without ciprofloxacin [5].

The 40% resistance of urinary pathogens towards the prior prophylactic agents is known in the literature, and can reach up to 60% [7,24]. Almost all scientific reports revealing the species of Gram-negative SSI in spine surgery (or orthopedic surgery in general) report a high proportion of non-fermenting rods among the Gram-negatives, which are naturally resistant to second-generation cephalosporins or vancomycin; and might also be selected by vancomycin powder that many spine surgeons administer immediately before wound closure at the end of the intervention [5,6,7,10,24,30,31,32]. According to experts, these (multi-resistant) bacilli have already started to colonize the perineum 2–3 days after admission [14].

Facing a possible link between perioperative UC use and SSI, the further question to address is the possibility of avoiding UC use during or after surgery, at least for the majority of patients. This is possible. Authors from Geneva proved the easy feasibility of a substantial reduction in perioperative UC use by 60% (with consecutive reduction in symptomatic UTI) among orthopedic patients, and its sustainability after eight years [33,34].

In case of postoperative urinary retention, a transient UC carriage is certainly of benefit, but routine UC use is not [1,2]. Indeed, a routine preoperative UC insertion does not seem to decrease postoperative bladder problems when compared to UC-free managements [2]. Similarly, the pre- or postoperative administration of antibiotics for asymptomatic urinary colonization has no preventive effects on SSIs in orthopedic surgery, but is associated with adverse events, costs, and carriage of antibiotic resistances [15,26,35,36,37]. In approximately half of our surgeries of both cohorts (2773 episodes), patients perioperatively carried a UC. This high proportion has the potential to be reduced.

Besides the retrospective design inherent to the study question, our study has three major limitations. First, patients with superficial SSIs may have been treated by the general practitioner [9,21]. However, as the authors’ institution is the only public university hospital for orthopedic spine surgery in Zurich and is following up with the patients over several years, we consider this bias as minimal. Second, we detected associations and cannot prove a direct causal relationship between a UC (or UTI) and Gram-negative SSIs. It could be that patients with Gram-negative SSI and/or UC use are simply sicker than the others. However, after case-mix adjustment by multivariate Cox regression analyses, we failed to associate specifically Gram-negative SSI to the patients’ underlying co-morbidities (in contrast to the occurrence of SSI in general). Only the duration of UC use and the female sex remained statistically associated with Gram-negative SSI, making a clinical relationship plausible. Third, we analyzed risk factors for SSI based on our experience and the established literature of spine infections, whereas the literature regarding the indications for UC use is much broader, including hemodynamic surveillance, past history of urinary retention, prolonged stay in the intensive care unit, massive blood loss, and behavioral science that might all influence the reason for UC insertion [2,13,14,21,23,24,38,39]. These “soft” indications often go unnoticed and are beyond the datamining capacity, and the consecutive statistical models, in a surgical clinic.

Formally, the microbiological congruences between UTI and SSI pathogens are based on the identification of the bacterial species and their antibiotic susceptibility patterns, which is an accepted method of comparison in daily clinical life. In our retrospective study, we cannot perform serotype analyses as would have been possible in a prospective trial. Lastly, the clinicians diagnosed the occurrence of UTI according to the clinical symptoms and the corresponding microbiological and laboratory results. Retrospectively, and in the aftermath of complicated spine surgeries in a multimorbid patient population, we cannot assess every different UTI symptom in its individual details and must rely on the medical and nursing records.

## 5. Conclusions

Gram-negative SSIs after spine surgery seem to be firmly associated with UC carriage beyond one day and with the female sex, according to the performed multivariate analysis. A weaker association might also exist between SSIs (any Gram) and prolonged UC carriage. In other words, a UC carriage might not only be a risk for nosocomial UTI, but might also be associated with early SSIs after spine surgery. This observation indicates careful indication for UCs in the perioperative period around spine surgeries [33,34].

## Figures and Tables

**Table 1:** Spine surgeries and patients associated with postsurgical urinary catheter use. Group comparison.

	No Catheter Use	Urinary Catheter	*p*-
n = 5485 surgeries	n = 2751	n = 2734	value *
Female sex	1265 (46%)	1541 (56%)	***0.0001***
Median age	56 years	68 years	***0.0001***
Median body mass index (BMI)	26.2 kg/m^2^	27.2 kg/m^2^	***0.0001***
Presence of diabetes mellitus	213 (8%)	398 (14%)	***0.0001***
Median ASA-Score	2 points	2 points	***0.2001***
Lumbar surgery	2317 (84%)	2103 (76%)	***0.0001***
Implant-related surgery	692 (25%)	1924 (69%)	***0.0001***
Revision surgery	597 (22%)	1164 (42%)	***0.0001***
Median operation time	67 min	128 min	***0.0001***
Median length of hospital stay (index surgery)	5 days	5 days	0.3057
Presence of a remote infection ^+^	1 (0%)	15 (1%)	***0.0001***

* Pearson χ^2^-test or Wilcoxon-ranksum-tests. Significant results (*p* < 0.05) are indicated in bold and italics; ^+^ Remote infection: every nosocomial infection besides the surgical site (e.g., urine, pneumonia, etc.).

**Table 2:** Group comparisons with all own surgeries (left); and between Gram-positive and Gram-negative surgical site infections (right), including all own SSIs (n = 67) and 97 external SSIs.

All Surgeries	No Infection	*p*-Value *	SSI	Gram+ SSI	*p*-Value *	Gram− SSI
	n = 5418		n = 67	n = 107		n = 27
Female sex	2753 (51%)	0.2210	29 (43%)	39 (36%)	***0.0001***	19 (70%)
Median age	63 years	0.1564	69 years	69 years	0.8460	68 years
Median body mass index	26.7 kg/m^2^	0.1431	27.9 kg/m^2^	27.2 kg/m^2^	***0.0464***	30.2 kg/m^2^
Active smoker	527 (28%)	***0.0007***	27 (44%)	-	-	-
Presence of diabetes mellitus	580 (11%)	***0.0001***	20 (30%)	23 (22%)	0.3700	8 (30%)
Presence of a remote infection	0 (0%)	***0.0001***	9 (13%)	11 (10%)	***0.0030***	9 (33%)
Median ASA-Score	2 points	***0.0001***	3 points	3 points	0.0528	3 points
ASA-Score > 3 points	78 (3%)	***0.0001***	5 (7%)	4 (4%)	***0.0421***	5 (19%)
Lumbar surgery	4332 (80%)	0.2791	50 (75%)	76 (71%)	0.2740	22 (81%)
Implant surgery	2564 (47%)	***0.0001***	18 (27%)	49 (46%)	0.6370	11 (61%)
Revision surgery	1672 (31%)	***0.0001***	58 (87%)	72 (68%)	0.3190	21 (78%)
Use of UC (yes/no)	2686 (50%)	***0.0001***	48 (72%)	-	-	-
Median UC use duration	0 day	***0.0001***	1 day	1 day	***0.0065***	3 days
Postoperative urinary cultures sampled	308 (6%)	***0.0001***	20 (30%)	-	-	-

* Pearson χ^2^-test or Wilcoxon-ranksum-tests. Significant results (*p* < 0.05) are indicated in bold and italics. “-” = external data not verifiable. “UC” = urinary catheter; “Gram+” = Gram-positive; “Gram−” = Gram-negative.

**Table 3:** Multivariate associations with the outcome “overall deep surgical site infection” SSI (left); Gram-negative SSIs (right). Cox regression analyses; results expressed as hazard ratios with 95% confidence intervals.

Occurrence of SSI	Univariate	Multivariate	Gram− vs. Gram+	Univariate	Multivariate
Female sex	0.7, 0.5–1.2	0.8, 0.4–1.3	Female sex	***4.1, 1.7–10.3***	***3.8, 1.4–10.6***
Age	1.0, 1.0–1.0	-	Age	1.0, 1.0–1.0	-
Body mass index	1.0, 0.9–1.1	-	Body mass index	***1.1, 1.1–1.2***	-
Diabetes mellitus presence	***3.5, 2.1–6.0***	***2.2, 1.1–4.2***	Diabetes mellitus presence	1.5, 0.6–4.0	-
Active smoking	***2.0, 1.2–3.3***	***2.4, 1.4–4.3***	Active smoking	-	-
ASA-Score	***2.9, 2.0–4.2***	***2.3, 1.4–3.6***	ASA-Score	***2.2, 1.1–4.5***	1.5, 0.6–3.9
Lumbar surgery	0.7, 0.4–1.3	-	Lumbar surgery	1.8, 0.6–5.2	-
Implant-related surgery	***1.4, 1.2–1.7***	-	Implant-related surgery	0.8, 0.3–1.9	-
Revision surgery	***14.4, 7.1–29.2***	***22.4, 9.8–51.2***	Revision surgery	1.7, 0.6–4.5	-
Use of any UC (yes/no)	***2.6, 1.5–4.4***	***2.5, 1.3–4.9***	Use of any UC (yes/no)	-	-
UC use for >1 day	***1.1, 1.1–1.1***	***1.1, 1.1–1.1***	UC use for >1 day	***4.5, 1.5–21.0***	***5.5, 1.5–20.3***
Urinary culture sampling	***6.9, 4.0–11.8***	-	Urinary culture sampling	-	-

Statistically significant results are displayed in bold and italics. “-” = not included in the multivariate model, data not sure, small sample size. “UC” = urinary catheter; “Gram+” = Gram-positive; “Gram−” = Gram-negative.

**Table 4:** Literature review in spine surgery. Arbitrary selection of published papers in line with our study objectives.

Reference	Year	Design	N.	Study Question	Results and Remarks
France [25]	2012 *	Observ.	169	Risk factors for SSI in spine trauma	UC use >5 days is associated with SSI. Only univariate analyses
Spain [5]	2014	Retro.	466	Relation between UTI and SSIs	Risk SSI 12%. Risk UTI 12%. 4% of SSI by the same germ as in UTI Odds ratio of UTI for SSI: 3.1; 95% CI 1.6–6.1
France [13]	2014 *	Observ.	518	Risk for SSI in spine trauma	UC use is significantly associated with SSI in group comparisons
Czech [21]	2021	Retro.	274	UC a risk for SSI	UC is not associated with lumbar SSI (91% vs. 96%; *p* = 0.5)
USA [26]	2022	Retro.	95	Postop. co-trimoxazole for 2 weeks	SSI 2.2% with antibiotic vs. 2.4% without (*p* = 0.9)

* Two studies stemming from the same center and the same research group. “N.” = number of patients; “Retro.” = Retrospective case-control study; “Observ.” = Prospective-observational; “UC” = Urinary catheter; “UTI” = Symptomatic urinary tract infection; “SSI” = Surgical site infection; “Postop.” = postoperative; “CI” = confidence interval.

## Data Availability

We may share anonymized variables upon reasonable scientific request to the corresponding author.

## References

[1] Crain N.A., Goharderakhshan R.Z., Reddy N.C., Apfel A.M., Navarro R.A. (2021). The Role of Intraoperative Urinary Catheters on Postoperative Urinary Retention after Total Joint Arthroplasty: A Multi-Hospital Retrospective Study on 9580 Patients. Arch. Bone Jt. Surg..

[2] Leitner L., Wanivenhaus F., Bachmann L.M., Liechti M.D., Aguirre J.A., Farshad M., Kessler T.M. (2021). Bladder management in patients undergoing spine surgery: An assessment of care delivery. N. Am. Spine Soc. J..

[3] Uckay I., Holy D., Betz M., Sauer R., Huber T., Burkhard J. (2021). Osteoarticular infections: A specific program for older patients?. Aging Clin. Exp. Res..

[4] Uçkay I., Sax H., Gayet-Ageron A., Ruef C., Mühlemann K., Troillet N., Petignat C., Bernasconi E., Balmelli C., Widmer A. (2013). High proportion of healthcare-associated urinary tract infection in the absence of prior exposure to urinary catheter: A cross-sectional study. Antimicrob. Resist. Infect. Control.

[5] Núñez-Pereira S., Rodríguez-Pardo D., Pellisé F., Pigrau C., Bagó J., Villanueva C., Cáceres E. (2014). Postoperative urinary tract infection and surgical site infection in instrumented spinal surgery: Is there a link?. Clin. Microbiol. Infect..

[6] Perry J.W., Montgomerie J.Z., Swank S., Gilmore D.S., Maeder K. (1997). Wound infections following spinal fusion with posterior segmental spinal instrumentation. Clin. Infect. Dis..

[7] Long D.R., Bryson-Cahn C., Pergamit R., Tavolaro C., Saigal R., Chan J.D., Lynch J.B. (2021). 2021 Young Investigator Award Winner: Anatomic Gradients in the Microbiology of Spinal Fusion Surgical Site Infection and Resistance to Surgical Antimicrobial Prophylaxis. Spine.

[8] Al Farii H., Slawaska-Eng D., Pankovitch S., Navarro-Ramirez R., Weber M. (2021). Gram-Negative Surgical Site Infections after 989 Spinal Fusion Procedures: Associated Factors and the Role of Gram-Negative Prophylactic Antibiotic Coverage. Int. J. Spine Surg..

[9] Uckay I., Hoffmeyer P., Lew D., Pittet D. (2013). Prevention of surgical site infections in orthopaedic surgery and bone trauma: State-of-the-art update. J. Hosp. Infect..

[10] Jamei O., Gjoni S., Zenelaj B., Kressmann B., Belaieff W., Hannouche D., Uçkay I. (2017). Which Orthopaedic Patients Are Infected with Gram-negative Non-fermenting Rods?. J. Bone Jt. Infect..

[11] Hasler A., Unterfrauner I., Olthof M.G., Jans P., Betz M., Achermann Y., Uçkay I. (2021). Deep surgical site infections following double-dose perioperative antibiotic prophylaxis in adult obese orthopedic patients. Int. J. Infect. Dis..

[12] Burkhard M.D., Loretz R., Uckay I., Bauer D.E., Betz M., Farshad M. (2021). Occult infection in pseudarthrosis revision after spinal fusion. Spine J..

[13] Dubory A., Giorgi H., Walter A., Bouyer B., Vassal M., Zairi F., Dhenin A., Grelat M., Lonjon N., Dauzac C. (2015). Surgical-site infection in spinal injury: Incidence and risk factors in a prospective cohort of 518 patients. Eur. Spine J..

[14] Galloway A. (1997). Prevention of urinary tract infection in patients with spinal cord injury—A microbiological review. Spinal Cord.

[15] Uçkay I., Lübbeke A., Emonet S., Tovmirzaeva L., Stern R., Ferry T., Assal M., Bernard L., Lew D., Hoffmeyer P. (2009). Low incidence of haematogenous seeding to total hip and knee prostheses in patients with remote infections. J. Infect..

[16] Sax H., Uckay I., Richet H., Allegranzi B., Pittet D. (2007). Determinants of good adherence to hand hygiene among healthcare workers who have extensive exposure to hand hygiene campaigns. Infect. Control Hosp. Epidemiol..

[17] Sax H., Allegranzi B., Uckay I., Larson E., Boyce J., Pittet D. (2007). ‘My five moments for hand hygiene’: A user-centred design approach to understand, train, monitor and report hand hygiene. J. Hosp. Infect..

[18] Uckay I., Sax H. (2006). “Bad news” for nosocomial infections. Krankenpfl. Soins Infirm..

[19] Farshad M., Bauer D.E., Wechsler C., Gerber C., Aichmair A. (2018). Risk factors for perioperative morbidity in spine surgeries of different complexities: A multivariate analysis of 1,009 consecutive patients. Spine J..

[20] Anderson P.A., Savage J.W., Vaccaro A.R., Radcliff K., Arnold P.M., Lawrence B.D., Shamji M.F. (2017). Prevention of Surgical Site Infection in Spine Surgery. Neurosurgery.

[21] Spatenkova V., Bradac O., Jindrisek Z., Hradil J., Fackova D., Halacova M. (2021). Risk factors associated with surgical site infections after thoracic or lumbar surgery: A 6-year single centre prospective cohort study. J. Orthop. Surg. Res..

[22] Meng F., Cao J., Meng X. (2015). Risk factors for surgical site infections following spinal surgery. J. Clin. Neurosci..

[23] Zhou J., Wang R., Huo X., Xiong W., Kang L., Xue Y. (2020). Incidence of Surgical Site Infection after Spine Surgery: A Systematic Review and Meta-analysis. Spine.

[24] Abdul-Jabbar A., Berven S.H., Hu S.S., Chou D., Mummaneni P.V., Takemoto S., Ames C., Deviren V., Tay B., Weinstein P. (2013). Surgical site infections in spine surgery: Identification of microbiologic and surgical characteristics in 239 cases. Spine.

[25] Lonjon G., Dauzac C., Fourniols E., Guigui P., Bonnomet F., Bonnevialle P., French Orthopaedic Surgery Traumatology Society (2012). Early surgical site infections in adult spinal trauma: A prospective, multicentre study of infection rates and risk factors. Orthop. Traumatol. Surg. Res..

[26] Choi J.H.K., Duong H.A., Williams S., Lee J., Oh M., Rosen C., Lee Y.P., Bhatia N. (2022). The efficacy of bactrim in reducing surgical site infections after spine surgery. N. Am. Spine Soc. J..

[27] Spina N.T., Aleem I.S., Nassr A., Lawrence B.D. (2018). Surgical Site Infections in Spine Surgery: Preoperative Prevention Strategies to Minimize Risk. Global Spine J..

[28] Atesok K., Papavassiliou E., Heffernan M.J., Tunmire D., Sitnikov I., Tanaka N., Rajaram S., Pittman J., Gokaslan Z.L., Vaccaro A. (2020). Current Strategies in Prevention of Postoperative Infections in Spine Surgery. Global Spine J..

[29] Galetta M.S., Kepler C.K., Divi S.N., Russo G.S., Segar A.H., Boody B.S., Bronson W.H., Rihn J.A., Goyal D.K., Fang T. (2020). Consensus on Wound Care of SSI in Spine Surgery. Clin. Spine Surg..

[30] Yoon J.W., Wanderman N.R., Kerezoudis P., Alvi M.A., De Biase G., Akinduro O.O., Berbari E.F., Bydon M., Freedman B.A. (2019). Enterobacter Infection after Spine Surgery: An Institutional Experience. World Neurosurg..

[31] Gande A., Rosinski A., Cunningham T., Bhatia N., Lee Y.P. (2019). Selection pressures of vancomycin powder use in spine surgery: A meta-analysis. Spine J..

[32] Lee Y.P., Farhan S.D., Pendi A., Cunningham T.J., Kiester P.D., Hahn P., Rosen C.D., Bhatia N. (2018). Does Addition of Tobramycin Powder Reduce Infection Rates after Spine Surgery?. Global Spine J..

[33] Stephan F., Sax H., Wachsmuth M., Hoffmeyer P., Clergue F., Pittet D. (2006). Reduction of urinary tract infection and antibiotic use after surgery: A controlled, prospective, before-after intervention study. Clin. Infect. Dis..

[34] Sax H., Kuster S.P., Tehrany Y.A., Ren R., Uçkay I., Agostinho A., Stephan F., Wachsmuth M., Walder B., Hoffmeyer P. (2016). Eight-year sustainability of a successful intervention to prevent urinary tract infection: A mixed-methods study. Am. J. Infect. Control.

[35] Bouvet C., Lübbeke A., Bandi C., Pagani L., Stern R., Hoffmeyer P., Uçkay I. (2014). Is there any benefit in pre-operative urinary analysis before elective total joint replacement?. Bone Jt. J..

[36] Uckay I., Lubbeke A., Huttner B. (2014). Preoperative asymptomatic bacteriuria and subsequent prosthetic joint infection: Lack of a causal relation. Clin. Infect. Dis..

[37] Sendi P., Borens O., Wahl P., Clauss M., Uckay I. (2017). Management of Asymptomatic Bacteriuria, Urinary Catheters and Symptomatic Urinary Tract Infections in Patients Undergoing Surgery for Joint Replacement: A Position Paper of the Expert Group ‘Infection’ of swissorthopaedics. J. Bone Jt. Infect..

[38] Uçkay I., Dinh A., Vauthey L., Asseray N., Passuti N., Rottman M., Biziragusenyuka J., Riché A., Rohner P., Wendling D. (2010). Spondylodiscitis due to Propionibacterium acnes: Report of twenty-nine cases and a review of the literature. Clin. Microbiol. Infect..

[39] Billières J., Uçkay I., Faundez A., Douissard J., Kuczma P., Suvà D., Zingg M., Hoffmeyer P., Dominguez D.E., Racloz G. (2016). Variables associated with remission in spinal surgical site infections. J. Spine Surg..

